# Understanding of how *Propionibacterium acidipropionici* respond to propionic acid stress at the level of proteomics

**DOI:** 10.1038/srep06951

**Published:** 2014-11-07

**Authors:** Ningzi Guan, Hyun-dong Shin, Rachel R. Chen, Jianghua Li, Long Liu, Guocheng Du, Jian Chen

**Affiliations:** 1 Key Laboratory of Carbohydrate Chemistry and Biotechnology, Ministry of Education, Jiangnan University, Wuxi 214122, China; 2 Key Laboratory of Industrial Biotechnology, Ministry of Education, Jiangnan University, Wuxi 214122, China; 3 School of Chemical and Biomolecular Engineering, Georgia Institute of Technology, Atlanta 30332, USA; 4 Synergetic Innovation Center Of Food Safety and Nutrition, Wuxi 214122, China

## Abstract

Propionic acid (PA) is an important platform chemical in the food, agriculture, and pharmaceutical industries and is mainly biosynthesized by propionibacteria. Acid tolerance in PA-producing strains is crucial. In previous work, we investigated the acid tolerance mechanism of *Propionibacterium acidipropionici* at microenvironmental levels by analyzing physiological changes in the parental strain and three PA-tolerant mutants obtained by genome shuffling. However, the molecular mechanism of PA tolerance in *P. acidipropionici* remained unclear. Here, we performed a comparative proteomics study of *P*. *acidipropionici* CGMCC 1.2230 and the acid-tolerant mutant *P*. *acidipropionici* WSH1105; MALDI-TOF/MS identified 24 proteins that significantly differed between the parental and shuffled strains. The differentially expressed proteins were mainly categorized as key components of crucial biological processes and the acid stress response. Quantitative reverse transcriptase polymerase chain reaction (qRT-PCR) was used to confirm differential expression of nine key proteins. Overexpression of the secretory protein glyceraldehyde-3-phosphate dehydrogenase and ATP synthase subunit α in *Escherichia coli* BL21 improved PA and acetic acid tolerance; overexpression of NADH dehydrogenase and methylmalonyl-CoA epimerase improved PA tolerance. These results provide new insights into the acid tolerance of *P*. *acidipropionici* and will facilitate the development of PA production through fermentation by propionibacteria.

Propionic acid (PA) is an important building block in organic synthesis, food, feedstuffs, perfume, paint, and pharmaceuticals^1^. PA is mainly produced by petrochemical synthesis. With increasing concerns about environmental pollution and energy shortages, there is growing interest in propionibacterial production of PA^2^,^3^. The highest reported PA production by bacteria was 136 g/L, obtained from constant fed-batch fermentation in a plant fibrous-bed bioreactor with immobilized *Propionibacterium freudenreichii* CCTCC M207015^4^. Despite these achievements, industrial-scale PA production has not been achieved due to the high production cost in comparison to traditional petrochemical-based production methods.

Microbial PA production is a typical product-inhibited process, and the severe inhibition of PA on cell growth and PA synthesis limits production. Enhancement of PA tolerance is an effective strategy to alleviate inhibition and improve PA production^2^,^3^. For example, fed-batch fermentation of *P*. *acidipropionici* mutants obtained by adaptation in a fibrous-bed bioreactor produced 71.8 g/L PA, 37.5% greater than the yield from the wild-type strain^2^. In previous work, we improved the acid tolerance of *P. acidipropionici* CGMCC 1.2230 by genome shuffling via inactivated protoplast fusion. The process yielded 3 PA-tolerant strains: *P*. *acidipropionici* WSH1103 (tolerance to 10 g/L PA), *P*. *acidipropionici* WSH1104 (tolerance to 15 g/L PA), and *P*. *acidipropionici* WSH1105 (tolerance to 20 g/L PA)^5^. To understand the mechanism and factors contributing to enhanced acid tolerance, the Yang group characterized the morphology of cells adapted in a fibrous-bed bioreactor^2^,^3^. The mutant strains were greater in length and smaller in diameter, giving them a higher surface area and making them more efficient in transporting substrates and metabolites across the cell membrane. Increased H^+^-ATPase activity in the mutant indicated greater proton pumping efficiency, which may improve PA tolerance. In addition, increased long-chain saturated fatty acids (C 17: 0) and fewer unsaturated fatty acids (C 18: 1) reduced membrane fluidity, which may also contribute to increased PA tolerance^2^,^3^. We recently investigated the acid-tolerance mechanism of *P*. *acidipropionici* at the microenvironmental level by comparing the physiological changes in wild-type *P*. *acidipropionici* and three mutants; we found that the arginine deaminase (ADI) and glutamate decarboxylase (GAD) systems are important for acid resistance in *P*. *acidipropionici*^6^. Despite these efforts, current understanding of the acid-tolerant mechanism of *P*. *acidipropionici* remains limited to the microenvironmental level, and it is not clear how *P*. *acidipropionici* responds to acid stress at the molecular level. This level of understanding is crucial, however, for engineering *P*. *acidipropionici* strains with improved acid tolerance and PA production.

To deepen our understanding of the adaptive response mechanism of *P*. *acidipropionici* to PA stress, we used comparative proteomics to characterize the differences between wild-type *P*. *acidipropionici* and the acid-tolerant mutant. First, 2-dimensional electrophoresis (2-DE)-based comparative proteomics was used to characterize the acid-tolerant mutants and identify differences in protein abundance relative to the parent strain. Proteins with significantly different expression patterns were verified at the transcriptional level by quantitative reverse-transcriptase polymerase chain reaction (qRT-PCR). Five key proteins were overexpressed in *Escherichia coli* BL21(DE3) to verify their ability to improve its tolerance to PA and acetic acid. The results deepen our understanding of the response of *P*. *acidipropionici* to PA stress and make it feasible to improve acid tolerance and PA production by direct genetic manipulation.

## Results

### Proteome analysis and protein identification

**Figure 1:**
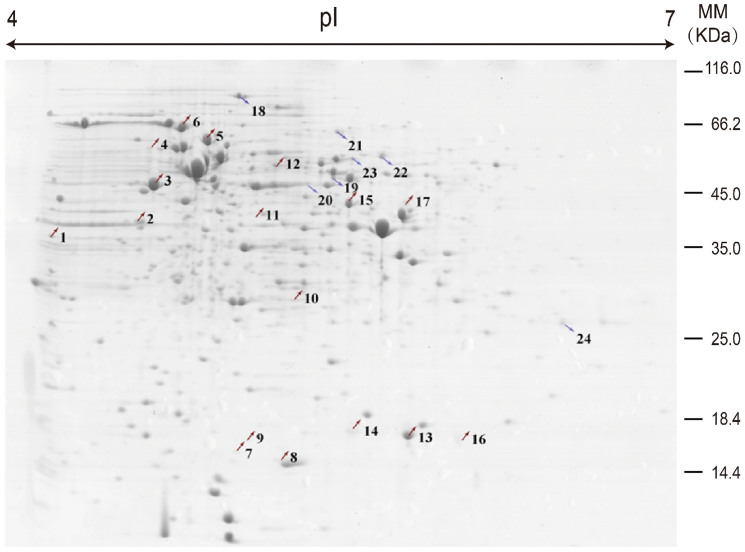
Comparative proteome map of *P*. *acidipropionici* CGMCC 1.2230 and *P*. *acidipropionici* WSH1105. (up arrow, the up-regulated proteins; down arrow, the down-regulated proteins.)

To better understand the molecular mechanism of *P. acidipropionici* acid tolerance, we used comparative proteomics to identify differential protein expression in *P. acidipropionici* CGMCC 1.2230 and its evolved mutant *P. acidipropionici* WSH1105 based on two-dimensional gel electrophoresis followed by protein identification. The gel maps (Figure S1) were analyzed with PDQuest 8.0.1. After optimization of the 2-DE gels, about 800 discrete intracellular protein spots were detected per sample. Proteins that differed by more than 2-fold were labeled in the preparative gel (Figure 1) and protein identities are listed in Table 1. Seventeen up-regulated and seven down-regulated protein spots were identified and are summarized in Table 1. The pIs of the protein spots ranged from 4.32 to 11.07, and the molecular masses ranged from 10.6 to 95.9 kDa. The theoretical and observed MW/pI values of the identified proteins were compared. A discrepancy between theoretical and observed MW/pI values was shown in our proteomics analysis. This discrepancy is a frequent phenomenon in proteome research and was mainly caused by post-translational modifications of proteins, such as proteolytic processing, glycosylation, and phosphorylation, or it was also possible that the protein spots detected were just protein fragments^7^,^8^. As shown in Table 1, the expression of 24 proteins was significantly different between the shuffled versus the parent strains. The differentially expressed proteins are classified into four categories: cellular metabolism and energy production (spots 2, 3, 4, 7, 8, 9, 10, 11, 12, 13, 14, 15, 18, 20, 22, 23, and 24); DNA replication, RNA synthesis, and translation (spot 1); posttranslational modification, protein turnover, and chaperones (spots 5, 6, 16, and 19); and hypothetical proteins of unknown function (spots 17 and 21).

### Verification of protein expression by qRT-PCRs

**Figure 2:**
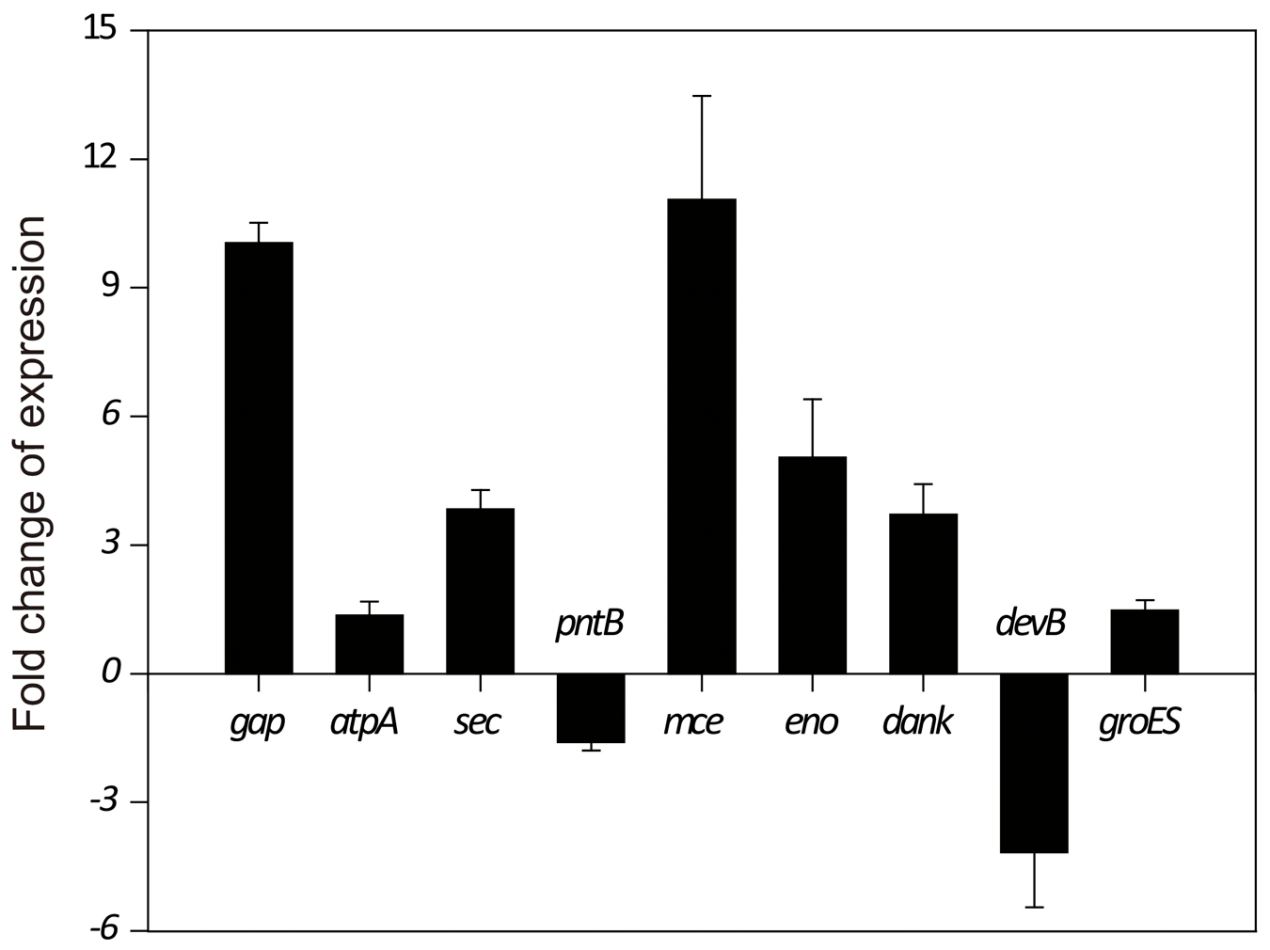
Changes of key genes transcription in *P*. *acidipropionici* WSH1105 compared with the parent strain *P*. *acidipropionici* CGMCC 1.2230. The values are from three independent cultivations.

Nine proteins with the greatest difference in expression between the parent and shuffled strains were verified by qRT-PCR. Glyceraldehyde-3-phosphate dehydrogenase, enolase, and 6-phosphogluconolactonase are key enzymes of central metabolism and crucial for growth. ATP synthase and NADH dehydrogenase mediate the balance of energy and oxidation-reduction, which is closely related to the stress response. Secretory protein transports substances and mediates the interactions between cells and the environment. Methylmalonyl-CoA is an important precursor of PA; thus, methylmalonyl-CoA epimerase may affect PA accumulation. These proteins most likely promote acid tolerance by modulating *P. acidipropionici* metabolism. The genes included glyceraldehyde-3-phosphate dehydrogenase (*gap*), ATP synthase subunit α (*atpA*), secretory protein (*sec*), NADH dehydrogenase (*pntB*), methylmalonyl-CoA epimerase (*mce*), enolase (*eno*), molecular chaperone Dnak (*dank*), 6-phosphogluconolactonase (*devB*), and heat shock protein GroES (*groES*). As shown in Figure 2, the transcription levels of 7 genes significantly increased in the shuffled strains, consistent with the proteomics results; however, the transcription levels of *pntB* and *devB* decreased in the shuffled strains, suggesting that the strains regulated the accumulation of NADH dehydrogenase and 6-phosphogluconolactonase at the level of translation not transcription.

### Overexpression of key proteins in *E. coli* BL21(DE3) to validate their function in acid tolerance

In order to examine the function of the identified proteins in acid tolerance, we developed the recombinant *E. coli* strains Ec-g (expressing glyceraldehyde-3-phosphate dehydrogenase), Ec-a (ATP synthase subunit α), Ec-s (secretory protein), Ec-p (NADH dehydrogenase), and Ec-m (methylmalonyl-CoA epimerase) and investigated their expression and effect on acid tolerance. Protein expression was estimated by measuring band intensity after separation by SDS–PAGE.

Acid tolerance was determined by viable-cell counting (Table 2, Figure S2). Ec-s exhibited the greatest tolerance to PA and acetic acid. In culture with 1 g/L PA, all recombinant strains showed better growth than the control Ec-1 (*E. coli* BL21(DE3) with pACYCDuet-1). Strains Ec-g and Ec-a survived in 2 g/L acetic acid., and only Ec-s exhibited tolerance to 2 g/L PA and 4 g/L acetic acid.

## Discussion

We performed a proteomics analysis of *P. acidipropionici* acid tolerance in wild-type *P. acidipropionici* CGMCC 1.2230 and the shuffled mutant *P. acidipropionici* wsh1105. The 24 differentially expressed proteins are involved in many crucial biological processes, and the abundance of these proteins varied significantly between the acid-tolerant and parent strains, indicating their importance for acid tolerance in *P. acidipropionici*. The detected proteins mainly belong to four functional classes: cellular metabolism and energy production; DNA replication, RNA synthesis, and translation; posttranslational modification, protein folding, and chaperones; and hypothetical proteins of unknown function.

The cell membrane offers first-level protection from various environmental pressures and is the first function to suffer damage in an acidic environment. Therefore, membrane proteins associated with acid tolerance in *P*. *acidipropionici* are particularly important. NADH-quinone oxidoreductase chain (NuoA) and ATP synthase subunit α (atpA) mediate membrane bioenergetics; their expression increased by 2.5- and 4.8-fold in the shuffled strains. As a multiple subunit enzyme complex embedded in the plasma membrane, NADH-quinone oxidoreductase pumps protons across the cytoplasmic membrane at the start of the respiratory chain and connects proton translocation (cytoplasmic to periplasmic phase) to electron transfer (NADH to quinone). The resulting membrane potential is used to drive processes such as ATP synthesis or solute transport^9^. Subunit NuoA is one of the membrane domain subunits that mediates H^+^ translocation. As shown in Table 1, the NADH-quinone oxidoreductase chain (NuoA) was significantly up-regulated in the shuffled strains, improving proton-pump capacity to adapt to the acidic environment.

In studies on *P*. *acidipropionici* acid tolerance at the microenvironment level, H^+^-ATPase, which is involved in proton-translocation, is critical to the maintenance of intracellular pH (pH_i_)^10^. The higher energy status in the shuffled strains provided more energy for H^+^-ATPase catalysis, leading to a higher pH_i_ than in the parental strain^6^. A 4.8-fold increase in atpA was observed in the shuffled strains, consistent with previous results in which the shuffled strains exhibited higher H^+^-ATPase activity and greater H^+^-ATPase synthesis capacity than the parental strain in order to maintain pH_i_ homeostasis.

**Figure 3:**
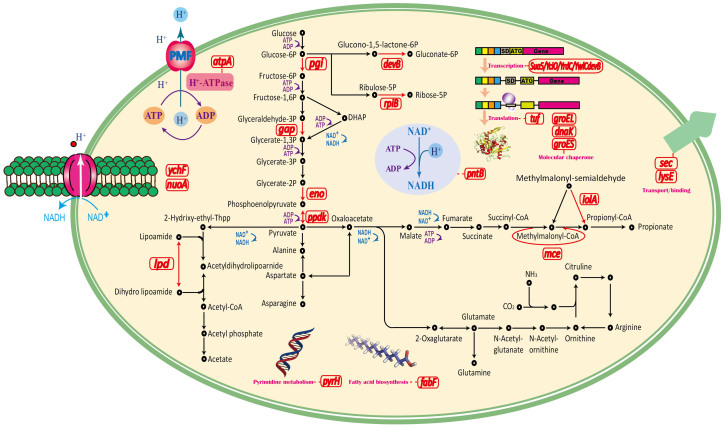
Overview of the differentially expressed proteins involved in the metabolism in *P*. *acidipropionici*.

The glycolytic pathway and TCA cycle play important roles in providing metabolic energy and intermediates during growth. Glucose can be converted into glucose-6-phosphate via the permease/glucokinase pathway and further metabolized into pyruvate by the glycolytic pathway (Figure 3). The differentially expressed proteins in the shuffled strains include glyceraldehyde-3-phosphate dehydrogenase, enolase, glucose-6-phosphate isomerase, ribose-5-phosphate isomerase, 6-phosphogluconolactonase, pyruvate phosphate dikinase, dihydrolipoamide dehydrogenase, and methylmalonyl-CoA epimerase. A 4.1-fold increase in glyceraldehyde-3-phosphate dehydrogenase indicated an increase in glycerate-1, 3-phosphate in the shuffled strains, while the levels of other intermediates are essentially the same, including glycerate-3-phosphate. Thus, the 5-fold increase in enolase and the 3.4-fold decrease in pyruvate phosphate dikinase improved the accumulation of phosphoenolpyruvate and pyruvate, which are used to generate PA. Methylmalonyl-CoA epimerase (MCE) catalyzes the reversible conversion of (S)-methylmalonyl-CoA to succinyl-CoA, which is a key intermediate in the TCA cycle. It is involved in the metabolism of propionate, branched-chain amino acids, and odd-chain fatty acids, where propionyl-CoA is converted to succinyl-CoA via (R,S)-methylmalonyl-CoA^11^. A 5.4-fold increase in MCE may partially explain the increased acid tolerance of the shuffled strains.

Glycolysis produces energy in the form of ATP as well as precursors for anabolic reactions. We previously showed that the metabolism of NAD^+^/NADH is vital for maintaining intracellular pH homeostasis^6^. Glyceraldehyde-3-phosphate dehydrogenase is a NAD^+^/NADH-dependent dehydrogenase that converts glyceraldehyde-3-phosphate to glycerate-1, 3-phosphate in glycolysis. Increased glyceraldehyde-3-phosphate dehydrogenase increases generation of NADH for reductive biosynthetic reactions. NADH dehydrogenase is directly involved in NAD^+^/NADH metabolism; a 3.7-fold increase in expression could significantly enhance the NAD^+^/NADH ratio and acid tolerance.

During the process of PA fermentation, pyruvate flows into two branches, leading to the formation of propionate or acetate (Figure 3). In the propionate branch of the pathway, 2 NADH molecules are oxidized to 2 NAD^+^; during reduction of pyruvate to acetate, an extra ATP is generated from ADP when acetyl phosphate is converted to acetate, before which acetyl-CoA is converted to acetyl phosphate as the previous step^12^. NADH oxidation can be readily adjusted to need by adjusting the two branches of the pathway, in which the oxidation-reduction balance of the cell is preserved. Methylmalonate-semialdehyde dehydrogenase is expressed at a lower level in the shuffled strains than in the parent strain; this enzyme catalyzes the conversion of 2-methyl-3-oxopropanoate to propanoyl-CoA and yields NAD^+^. It also converts 3-oxopropanoate to acetyl-CoA as a participant of the other branch of pyruvate. Thus, the alterations in methylmalonate-semialdehyde dehydrogenase and NADH dehydrogenase help maintain redox balance during fermentation.

Amino acids are important to the acid tolerance of *P. acidipropionici*. The ADI and GAD acid-resistant systems protect cells against the damaging effects of acidic environments^6^. As central metabolic pathways, the glycolytic pathway and TCA cycle produce precursors for many compounds, including some amino acids. The proteins identified in this study are directly or indirectly involved in amino acid metabolism (Figure 3). As a transport protein, LysE exports l-lysine, thereby regulating the intracellular concentration of amino acid. A thorough analysis revealed that LysE is a member of the transmembrane solute translocators^13^. Secretory protein also transports or binds proteins and lipoproteins. To ensure normal cellular activity in an acidic environment, more synthetized proteins need to be removed from the cells. Thus, the 2.5- and 3.1-fold increase in the accumulation of LysE and secretory protein may also enhance acid tolerance. GTPase YchF belongs to a group of universally conserved bacterial GTPases; only a few studies have investigated its function in bacteria. Many bacterial GTPases function by transmitting signals from the ribosome to downstream effectors for specific cellular responses^14^. GTPase YchF of *P*. *acidipropioni*ci may play an important role in the regulation of acid tolerance.

Acid stress has a major impact on protein synthesis^15^. Five proteins were associated with stress-response pathways involved in transcription, translation, and protein folding. Under acidic conditions, the DNA repair and protection responses needed for cell survival require the transcription of appropriate genes. Acid exposure in the shuffled strains induced a 2.5-fold increase in the expression of the transcription regulator Sua5/YciO/YrdC/YwlC. Further research is needed to understand its function in acid tolerance.

Acid exposure also affected the expression of elongation factor Tu (EF-Tu), which is used to transport aminoacyl tRNAs to the ribosome and to translocate the ribosome down the mRNA^16^. EF-Tu behaves as a chaperone for unfolded and denatured proteins in *E. coli*, preventing aggregation of citrate synthase during heat shock and producing stable complexes with some unfolded proteins (e.g., reduced carboxymethyl alpha-lactalbumin)^18^. A 2.7-fold decrease in EF-Tu was observed in the shuffled strains, consistent with previous reports that EF-Tu may mediate protein folding and protection during stress in addition to its function in translation elongation^17^.

EF-Tu recognizes the same hydrophobic binding motifs as the chaperone DnaK^18^, which was also induced in the shuffled strains. DnaK in *P. freudenreichii* was reported as a universal chaperone induced by bile salts^19^. The DnaK chaperone machinery prevents misfolding and aggregation of ribosome-bound polypeptides^16^,^20^ and is part of the heat-shock response-system^21^. DnaK up-regulation under acid stress was observed previously^22^. GroEL and GroES are chaperonins known as heat shock-induced promoters, which are essential for bacterial growth. The GroEL/GroES protein folding chaperonin complex is formed and dissociated by ATP binding and hydrolysis^23^. GroEL assists the folding of stress-denatured polypeptides by actions of binding and encapsulation^24^. It is induced during acid adaptation in *P. freudenreichii*^19^. Proteome analysis showed 3.1- and 4.3-fold up-regulation of GroEL and GroES, respectively, in the shuffled strains. Similar results have been reported in which groEL mRNA expression was increased 2.5-fold by acid shock with change in pH from 7.0 to pH 5.0^25^.

Beta-ketoacyl-[acyl-carrier-protein] synthase FabF was 3.0-fold down-regulated in the shuffled strain. Beta-ketoacyl-[acyl-carrier-protein] synthases are necessary for a Type II dissociated fatty acid biosynthetic system. Multiple isoforms of the synthase have been identified; synthase II has a lower pH optimum and greater resistance to stress^26^. FabF participates in the chain-elongation step of dissociated fatty-acid biosynthesis, during which post-translational modification is required for carrier proteins via phosphopantetheinyl transferase to achieve covalent attachment of the acyl intermediates to acyl carrier protein^27^. FabF regulates fatty-acid composition with changes in growth conditions and is associated with bacterial stress response^28^. Fatty acid distribution has been studied as a common mechanism utilized by *L. casei* to withstand severe acidification and to reduce the deleterious effect of lactic acid on the cell membrane^29^. It seems that microorganisms attempt to maintain normal metabolism in the face of environmental fluctuations by changing the lipid composition of their membranes. Down-regulation of FabF changed membrane fatty acid composition by adjusting the ratio of unsaturated to saturated fatty acids (U/S) and mean chain length, increasing membrane fluidity and permeability of the shuffled strain. This is vital to maintaining homeostasis and effective transmembrane processes such as nutrient transport or solute gradients for energetic purposes.

UMP kinase is specific to uridine monophosphate and is used in the de novo synthesis of pyrimidines. As the reaction it catalyzes is an ATP-consuming process, ATP seems to be economized to reduce the pernicious effects of acid via 2.1-fold down-regulation in the shuffled strains.

Plasmids have not been developed for gene expression in *P. acidipropionici*; therefore, overexpression of key proteins was conducted in *E. coli* BL21(DE3). Acid tolerance is an important property of *E. coli*, enabling the organism to survive gastric acidity and volatile fatty acids produced in the intestine^30^. *E. coli* is commonly used to produce various organic acids or other products with organic acid by-products. Thus, many studies have described and improved the acid tolerance mechanisms in *E. coli*; these studies involved transcriptome analysis, directed evolution, and overexpression and deletion of related proteins^31^,^32^,^33^. Several genes that function in multidrug transporter membrane ATP-binding components, DNA biosynthesis, replication, and repair, transcriptional regulator, and GTPase have been found to serve as acid resistance genes, similar to our findings. In this study, five proteins (glyceraldehyde-3-phosphate dehydrogenase, ATP synthase subunit α, secretory protein, NADH dehydrogenase, and methylmalonyl-CoA epimerase) were overexpressed in *E. coli* BL21(DE3); all yielded varying levels of PA or acetic acid tolerance. Secretory protein overexpression significantly improved the tolerance of *E. coli* BL21 to PA and acetic acid. *E. coli* BL21 expressing glyceraldehyde-3-phosphate dehydrogenase and ATP synthase subunit α grew well in low-concentration PA and acetic acid; *E. coli* overexpressing NADH dehydrogenase and methylmalonyl-CoA epimerase tolerate only 1 g/L PA. These improvements in acid tolerance ensure the metabolic processes of *E. coli* will not be inhibited by acidic products, and help cells overcome the damage caused by an acidic environment.

Ours is the first proteomic study of acid tolerance in *P. acidipropionici* and the differentially expressed proteins involved in many crucial biological processes. qRT-PCR was performed to verify nine proteins that exhibited the greatest differences between the parent and shuffled strains; transcription of seven of these genes significantly increased, consistent with the proteomic findings. Overexpression of five key proteins in *E. coli* BL21(DE3) validated their function in acid tolerance and the recombinant *E. coli* may have utility as fermentation strains. These results provided insights into the acid-tolerance mechanism of *P. acidipropionici* and enhanced bacterial survival under stress and inform the development of improved PA-producing strains.

## Methods

### Microorganisms and culture conditions

The parent strain *P. acidipropionici* CGMCC 1.2230 was purchased from the China General Microbiological Culture Collection (CGMCC). *P. acidipropionici* WSH1105 tolerates 20 g/L PA was obtained by genome shuffling^5^. The strains were inoculated at 1% (v/v) into 100-mL anaerobic jars containing 100 mL sterile medium (10 g/L yeast extract, 5 g/L tryptic soy broth, 1.5 g/L KH_2_PO_4_, and 2.5 g/L K_2_HPO_4_, pH 7.0). The anaerobic jars were sealed with butyl rubber caps and incubated for 48 h at 30°C.

### Protein extraction

Proteins were extracted from 15-mL aliquots. Cells were harvested in mid-exponential phase and collected by centrifugation (10,000 × *g*, 5 min). After washing 3 times with ultrapure water, the cells were lysed in 1 mL buffer containing 7 M urea, 2 M thiourea, 4% 3-[(3-cholamidopropyl)dimethylammonio]propanesulfonate (CHAPS), and 1% protease inhibitor, and the solution was sonicated on ice for 40 min, followed by centrifugation at 20,000 × *g* for 30 min. The proteins in the supernatant were treated with the Clean-up Kit (GE Healthcare) and dissolved in rehydration buffer (7 M urea, 2 M thiourea, 4% CHAPS, 1% (w/v) dithiothreitol (DTT), 0.5% (v/v) biolytes pH range 3–10 (Bio-Rad), 0.001% (w/v) bromophenol blue). Protein concentration was determined using the BioRad Protein Assay Kit (Bio-Rad) with BSA standard.

### Two-dimensional gel electrophoresis

Isoelectric focusing (IEF) of the protein samples was performed on immobilized pH gradient (IPG) strips (24 cm, 4–7 pH linear gradient; Bio-Rad). Samples were diluted to the same concentration with rehydration buffer (400–800 μg protein/450 μL). IPG strips were passively rehydrated for 14 h by soaking in 450 μL protein samples at room temperature. IEF was performed with Ettan™ IPGphor™ 3 (GE Healthcare) using a linear ramp program: 0 to 50 V in 30 min, 50 to 150 V in 1 h, 150 to 500 V in 1 h, 500 to 1000 V in 1 h, 1000 to 2500 V in 1 h, 2500 to 5000 V in 3 h, and 5000 to 10000 V in 3.5 h with rapid ramping to 90,000 Vhr. The temperature was maintained at 20°C and the electric current limit was set to 50 μA/gel. Before the second dimension, consecutive equilibrations of the gels in equilibration buffer (6 M urea, 2% sodium dodecyl sulfate (SDS), 0.375 M Tris-HCl, 20% glycerol, 0.002% bromophenol blue, pH 8.8) containing 0.8% (w/v) DTT and 1% (w/v) iodoacetamide were performed for 15 min as suggested by Görg et al.^34^.

Second-dimension electrophoresis was performed on an Ettan DALTsix Electrophoresis System (GE Healthcare). After rinsing in Tris-glycine electrode buffer (25 mM Tris, 192 mM glycine, 0.1% SDS, pH 8.3), the IPG strips were placed on 1-mm-thick SDS (12.5% (w/v)) polyacrylamide gels and sealed with 1% (w/v) agarose containing a trace of bromophenol blue. SDS electrophoresis was performed first at 2 W/gel for 90 min, and then at 14 W/gel until the dye front was 1 mm from the bottom of the gel. The temperature was maintained at 12.5°C with a MultiTemp III system (GE Healthcare).

### Staining and image analysis

Gels were fixed in fixation fluid (80% methanol, 20% acetic acid) for 30 min and stained overnight with Coomassie blue (1.2 g/L Coomassie G-250, 100 g/L (NH_4_)_2_SO_4_, 100 g/L phosphoric acid, 20% methanol). Gels were scanned and images captured with Image Master LabScan (GE Healthcare) after washing in ultrapure water until the background was colorless. Three biological duplications for each sample were tested as described. The gel images were analyzed using the PDQuest 8.0.1 software package (Bio-Rad), including image filtering, background subtraction, normalization, and spot detection^35^. Manual determination of spot positions was performed after automatic matching. Spots with quantitative changes (text/control) >2 or <0.5 were considered.

### Protein identification by mass spectrometry

Spots of interest were excised from the preparative gels and transferred to microtubes. Spots were digested with bovine trypsin (sequencing grade Roche Molecular Biochemicals) using an Ettan™ digester station (GE Healthcare Life Sciences) as described by Jun et al.^36^, with minor variations. Briefly, spots were washed twice with water and destained by 2 × 10 min incubation in 100 mM ammonium bicarbonate and 30% acetonitrile (v/v) and dried in a Savant SpeedVac for 30 min. The samples were immersed in 5 μL of trypsin (10 ng/μL) in 25 mM ammonium bicarbonate (pH 8.0) at 4°C for 60 min and the remaining liquid was removed. Then, samples to which 20 μL 25 mM ammonium bicarbonate (pH 8.0) had been added were digested overnight at 37°C. After digestion, 100 μL TA60 (mixture containing 60% ACN and 0.1% TFA) were added to the spots and merged with the former enzymatic hydrolysate. Finally, the mixture was concentrated to 5 μL; 1 μL of this and 0.4 μL of a 3 mg/mL α-cyano-4-hydroxy-transcinnamic acid matrix in TA85 (mixture containing 85% ACN and 0.1% TFA) were spotted onto a MALDI target plate using the dry droplet method for protein identification by mass spectrometry^37^. The peptide fragment ion data acquired from Bruker UltraFlex III MALDI-TOF/TOF mass spectrometer (Bruker Daltonics, Karlsruhe, Germany) were used to search for protein candidates in the NCBInr database through Mascot (Matrix Science) integrated in Biotools (Bruker Daltonics), which is accessible online. Scores calculated by the Mowse scoring algorithm in MASCOT (*p* < 0.05) were considered as positive identifications. The following search parameters were used: the peptide ions and MS/MS tolerance were set to 300 ppm and 0.9 Da respectively; carbamidomethyl and oxidized methionine were set as fixed and variable modifications, respectively, and 1 missed cleavage was allowed. The identified proteins were divided into different categories by the information provided in the NCBInr database.

### RNA isolation and quantitative reverse transcription-PCR (qRT-PCR)

Cells were harvested in mid-exponential phase and immediately transferred into liquid nitrogen to block the metabolism. Total RNA was extracted using an RNAisoPlus kit (TaKaRa, Dalian, China), and quantitation was performed at 260/280 nm in a Nanodrop ND-2000 spectrophotometer (Thermo Scientific, Wilmington, DE, USA). cDNA was synthesized using PrimeScript RT reagent Kit Perfect Real Time (TaKaRa Bio-Inc, Otsu, Shiga Japan). The qRT-PCR was conducted in a LightCycler 480 II Real-time PCR instrument (Roche Diagnostics, Mannheim, Germany) and performed using a SYBR Premix Ex Taq Kit (TaKaRa Bio-Inc, Otsu, Shiga Japan) with a 20.0 μL system: 10.0 μL SYBR *Premix Ex Taq* (2×), 0.4 μL PCR Forward Primer (10 μM), 0.4 μL PCR Reverse Primer (10 μM), 1.2 μL DNA template, 8 μL dH_2_O. The parameters were: pre-incubation at 95°C for 30 s; 40 cycles of amplification step 95°C for 5 s, 60°C for 20 s; cooling at 50°C for 30 s. The qRT-PCR gene-specific primers designed in Primer Express 2.0 (Table 3). 16S rRNA processing protein (*rimM*) was used as a reference gene for normalization. All experiments were performed with at least three biological replicates.

### Plasmid and strain construction

*E. coli* JM109 was used for plasmid cloning and maintenance; *E. coli* BL21(DE3) was used as a host for protein expression. Luria-Bertani (LB) medium was used for routine culture maintenance. Chloramphenicol 25 μg/mL was added where indicated. Gene manipulations were carried out using standard methods. In *P. acidipropionici*, *gap* (lp_1005), *atpA* (lp_1707), *sec* (lp_1152, named by author), *pntB* (lp_1413), and *mce* (lp_447, named by author) encode glyceraldehyde-3-phosphate dehydrogenase, ATP synthase subunit α, secretory protein, NADH dehydrogenase, and methylmalonyl-CoA epimerase, respectively. *cpr* (lp_1938, 71.5 KDa), which encodes cytochrome P450 reductase from *Catharanthus roseus* and is not related to acid stress resistance, was used as the control^38^. The genes were cloned into pACYCDuet-1 vector with the T7 promoter. To overexpress glyceraldehyde-3-phosphate dehydrogenase, the *gap* coding region was PCR-amplified from the genomic DNA of *P. acidipropionici* and cloned into pACYCDuet-1 at the *Nco*I and *Hind*III sites to yield pACYC-*gap*. pACYC-*atpA*, pACYC-*sec*, pACYC-*pntB*, pACYC-*mce*, and pACYC-*cpr* were constructed by inserting *atpA* between *Nde*I and *Eco*RV, *sec* between *Nco*I and *Hind*III, *pnt*B between *Nde*I and *Kpn*I, *mce* between *Nco*I and *Not*I, and *crp* between *Nco*I and *Not*I. PCR was performed using primers designed in Primer 5 (Table S1). Gene insertion was verified by restriction mapping, and the absence of undesired mutations introduced during PCR was verified by direct nucleotide sequencing.

### Acid tolerance analysis

Viable-cell counts were used to determine whether gene overexpression enhanced the acid tolerance of *E. coli*. For protein expression, overnight pre-inocula were prepared in LB medium at 37°C and transferred to fresh LB at a starting A_600_ of 0.1 and grown to A_600_ 0.6. Expression of the recombinant genes was induced by addition of 1 mM IPTG and incubation continued at 30°C for an additional 2 h. Cultures were then challenged with 1 g/L PA, 2 g/L PA, 2 g/L acetic acid, and 4 g/L acetic acid. Incubation continued at 30°C to stationary phase and cell survival was analyzed by viable-cell counting. At this stage, the density of the *E. coli* cultures was about 10^8^ cells/mL; 1 mL of the same culture was added to 9 mL distilled water, and several 10-fold dilutions were made and spread on agar plates. The plates were incubated at 37°C and colonies were counted after 12 h.

### Statistical analysis

All experiments were performed at least three times, and the results are expressed as the mean ± standard deviation (n = 3). Data were analyzed using the Student's t test. P values less than 0.05 were considered statistically significant.

## Author Contributions

L.L., G.C.D. and J.C. conceived and designed the experiments; N.Z.G. performed the experiments; H.D.S. and R.C. analyzed the data; N.Z.G., J.H.L. and L.L. wrote the paper.

## Supplementary Material

Supplementary Information Supplementary figures and tables

## Figures and Tables

**Table 1:** Identification of proteins between *P*. *acidipropionici* CGMCC 1.2230 and three genome-shuffled strains

					Mass(Da)/pI^3^		FoldChange^4^	
Putative function	Spot no.^1^	Protein	Gene	Mascot score	Theoretical	Observed		p-value
Transcription regulation	1	Sua5/YciO/YrdC/YwlC		69	29253/4.32	34990 ± 230/4.19 ± 0.02	2.5	0.033
Metabolism of coenzymes and prosthetic groups	2	Glyceraldehyde-3-phosphate dehydrogenase	*gap*	156	36110/5.26	37510 ± 370/4.58 ± 0.05	4.1	0.035
	7	NADH dehydrogenase	*pntB*	61	49342/5.08	15440 ± 320/5.08 ± 0.00	3.7	0.041
Main glycolytic pathways	3	Enolase	*eno*	121	45947/4.47	45940 ± 0.0/4.66 ± 0.10	5.0	0.003
	10	Glucose-6-phosphate isomerase	*pgi*	76	61876/5.16	28750 ± 330/5.16 ± 0.00	2.8	0.019
	11	Ribose-5-phosphate isomerase	*rpiB*	60	15427/5.61	38090 ± 220/5.11 ± 0.03	3.2	0.026
	15	6-phosphogluconolactonase	*devB*	95	26595/4.93	42960 ± 330/5.54 ± 0.07	4.9	0.045
Membrane bioenergetics (electron transport chain and ATP synthase)	4	GTPase YchF	*ychF*	98	39312/4.64	55500 ± 180/4.64 ± 0.00	2.2	0.035
	12	ATP synthase subunit alpha	*atpA*	520	61373/5.06	52630 ± 230/5.21 ± 0.17	4.8	0.038
	13	NADH-quinone oxidoreductase chain	*nuoA*	382	31310/7.17	16620 ± 170/5.85 ± 0.09	2.5	0.047
Protein folding	5	Molecular chaperone GroEL	*groEL*	124	56157/4.72	56150 ± 0.0/4.72 ± 0.00	3.1	0.026
	6	Molecular chaperone DnaK	*dnaK*	76	67224/4.66	65880 ± 40/4.66 ± 0.00	5.0	0.051
	16	Heat shock protein 10	*groES*	195	10596/4.95	16340 ± 500/6.06 ± 0.31	4.3	0.027
Transport/binding of amino-acids	8	Lysine exporter protein	*lysE*	66	21110/11.07	14510 ± 270/5.24 ± 0.08	2.5	0.046
Transport/binding proteins and lipoproteins	9	Secretory protein	*Sec*^2^	89	40255/5.27	15610 ± 190/5.10 ± 0.14	3.1	0.007
Specific carbohydrate metabolic pathway	14	Methylmalonyl-CoA epimerase	*mce*^2^	164	16715/5.41	16720 ± 0.0/5.57 ± 0.05	5.4	0.038
	22	Methylmalonate-semialdehyde dehydrogenase	*iolA*	145	52823/5. 00	52820 ± 0.0/5.70 ± 0.08	0.30	0.014
Protein of unknown function	17	Hypothetical protein		75	59417/4.93	40180 ± 2040/5.81 ± 0.13	2.7	0.030
	21	Hypothetical protein		126	58802/10.52	64020 ± 1800/5.49 ± 0.05	0.31	0.027
Metabolism of carbohydrates and related molecules	18	Pyruvate phosphate dikinase	*ppdk*	111	95874/4.81	94820 ± 190/5.03 ± 0.07	0.29	0.014
	23	Dihydrolipoamide dehydrogenase	*Ipd*	79	49613/5.21	52820 ± 240/5.55 ± 0.06	0.31	0.037
Translation elongation	19	Elongation factor Tu	*tuf*	91	43677/5.05	46660 ± 70/5.45 ± 0.30	0.37	0.044
Metabolism of lipids	20	Beta-ketoacyl-ACP synthase	*fabF*	95	43714/5.07	46050 ± 140/5.34 ± 0.09	0.33	0.042
Metabolism of nucleotides and nucleic acids	24	UMP kinase	*pyrH*	82	27041/4.84	27040 ± 0.0/6.50 ± 0.06	0.48	0.025

^1^Spot numbers refer to the proteins labeled in Figure 1.

^2^The two genes were named by author.

^3^Discrepancys exist between the measured and the predicted ones because of the modifications and degradations.

^4^The values were fold change of proteins in shuffled strain to parental strain.

**Table 2:** Comparison of the survival rates (%) between *E. coli* BL21 with pACYCDuet-1 (Ec-1) and the recombinant strains in propionic acid and acetic acid

	1 g/L propionic acid	2 g/L propionic acid	2 g/L acetic acid	4 g/L acetic acid
Ec-1	0.61	0.35	0.015	<0.003
Ec-c	0.72	<0.003	0.010	<0.003
Ec-g	100	0.1	0.023	<0.003
Ec-a	100	0.043	17	<0.003
Ec-s	100	100	2.86	0.043
Ec-p	3.57	0.008	<0.003	<0.003
Ec-m	7.61	<0.003	<0.003	<0.003

(Each value represents the mean of triple independent measurements, and the deviation from the mean was below 10%.).

**Table 3:** Primers used in the qRT-PCR

	Sequence Length	Product Length	Sense Primer (5′-3′)	Anti-sense Primer (5′-3′)
*rimM*	552	197	GTGGTGTCCGATGTGCTC	TGCTTGCTTCCCGTGTTG
*gap*	1005	145	CACTGATGGCGAGAAGGC	GCTGATGATGTTGTGCTTGG
*pntB*	1413	125	TGATCCTCGGTGGCTTCG	GGTGATGATGACGCTCTCG
*atpA*	1707	145	CACCACCATCGCATCCAG	CTCATTGCCGACCTTCTCC
*sec*	1152	183	GATGCCGCTGTGTTGTCC	CTCGTCGTCGCTGTCAAAG
*mce*	447	134	CGACGAGGCTTCCAAGTAC	GAACCTGGGTCATGTGCTC
*eno*	1290	192	CTCAAGGGCGTGCTCAAG	GCCATCGGTGTAGAACTCC
*dnaK*	1875	153	ACCACCGACATCAACATTCC	CTTGGCGTCCTTCAGCAC
*devB*	744	90	ACGAGGAGTTGACCACAGG	ACAGGCAGAGGTTCACATATTC
*groES*	297	165	AAGGAGAAGCCGCAGGAG	GTTGAGCAGCAGGTAGTCG
