# Supplementary material for: Understanding of how Propionibacterium acidipropionici respond to propionic acid stress at the level of proteomics
Source: Sci Rep. 2014 Nov 7;4:6951. doi: 10.1038/srep06951 (PMC4223659; doi:10.1038/srep06951)
Supplement: Supplementary Information — Supplementary figures and tables [file srep06951-s1.pdf]

Supplementary Information

Understanding of how *Propionibacterium acidipropionici* respond to propionic acid stress at the level of proteomics

Ningzi Guan[1, 2], Hyun-dong Shin[3], Rachel R. Chen[3], Jianghua Li[1, 4], Long Liu[1,4*], Guocheng Du[4*], Jian Chen[4]

[1]Key Laboratory of Carbohydrate Chemistry and Biotechnology, Ministry of Education, Jiangnan University, Wuxi 214122, China

[2]Key Laboratory of Industrial Biotechnology, Ministry of Education, Jiangnan University, Wuxi 214122, China

[3]School of Chemical and Biomolecular Engineering, Georgia Institute of Technology, Atlanta 30332, USA

[4]Synergetic Innovation Center Of Food Safety and Nutrition, Wuxi 214122, China

Corresponding authors: Long Liu, Tel.: +86-510-85918312, Fax: +86-510-85918309, E-mail: longliu@jiangnan.edu.cn; Guocheng Du, Tel.: +86-510-85918309, Fax: +86-510-85918309, E-mail: gcdu@jiangnan.edu.cn.

Table S1: Primers used in PCR for recombinant strains construction

	**Sense Primer (5'-3')**	**Anti-sense Primer (5'-3')**
gap	CATGCCATGGCAATGACCGTCAAGGTTGGTAT	CCCAAGCTTTCAGAGCTTGTCGGCAAC
sec	CATGCCATGGCAGTGCGCGACGATGACCTTG	CCCAAGCTTTCATGGCGTCACCCCCG
atpA	GGGAATTCCATATGGCCGACAAGTTACCC	GGAAGATATCTCAGGACCTCTTCGTGGC
pntB	GGGAATTCCATATGGTGCTCCTCGAAACTGATT	CGGGGTACCCTACTTGGCGTCGGCCGT
mce	CATGCCATGGGCATGAGTAATGAGGATCTTTTCATC	AAGGAAAAAAGCGGCCGCTCAGTTCTTCGGGTACTGG
cpr	CATGCCATGGGCAGCAGTGGCAGTGGCAAG	AAGGAAAAAAGCGGCCGCTCACCACACATCACGCAGAT

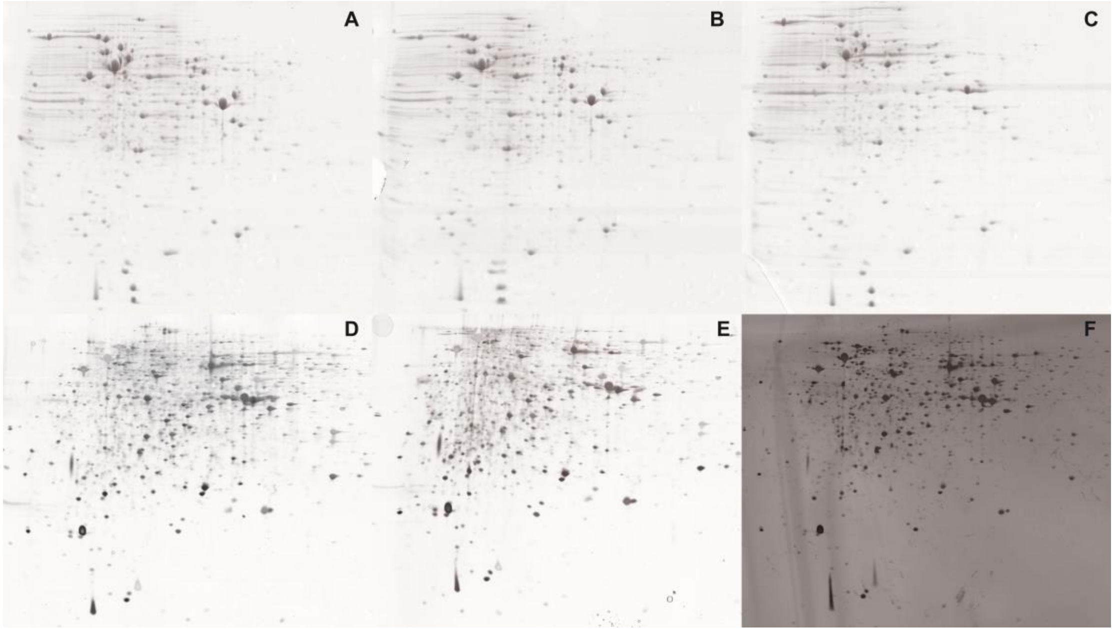

Figure S1: Protein profile of the parental strain (A-C) and the shuffled strain (D-F) on the gel.

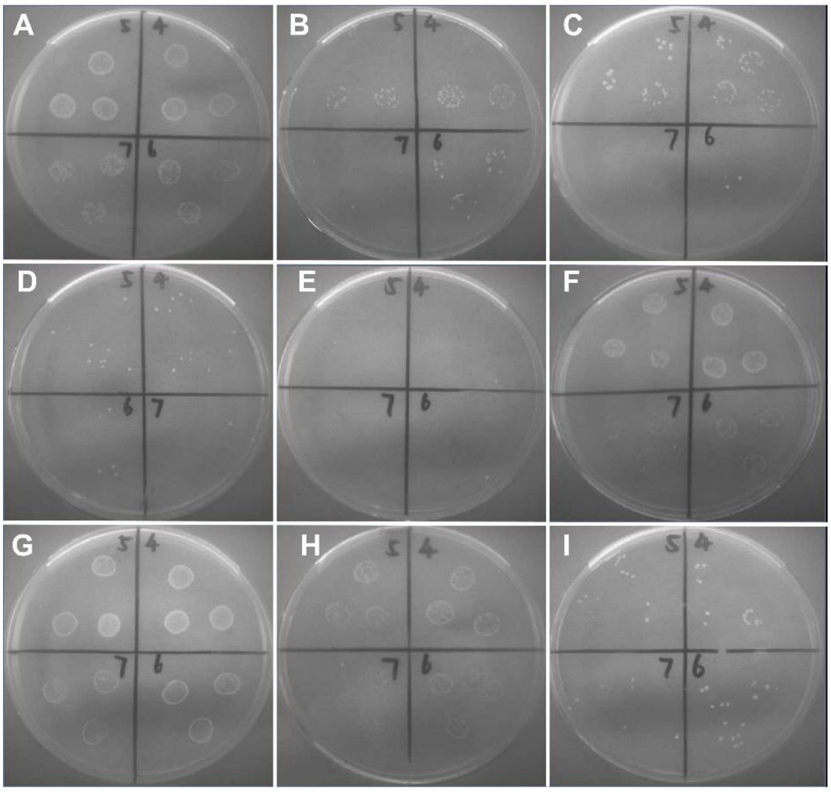

Figure S2: A portion of the culture dishes of recombinant strains grown with additional acids used in viable-cell counting. (A. Ec-1, without acid; B. Ec-c, 1 g/L propionic acid; C. Ec-1, 2 g/L propionic acid; D. Ec-a, 2 g/L propionic acid; E. Ec-p, 4 g/L acetic acid; F. Ec-g, 1 g/L propionic acid; G. Ec-s, 1 g/L propionic acid; H. Ec-s, 2 g/L propionic acid; I. Ec-m, 1 g/L propionic acid)
